# Supplementary material for: Dissecting the genetic landscape of GPCR signaling through phenotypic profiling in C. elegans
Source: Nat Commun. 2023 Dec 18;14:8410. doi: 10.1038/s41467-023-44177-z (PMC10728192; doi:10.1038/s41467-023-44177-z)
Supplement: Supplementary file 1 — Supplementary Information [file 41467_2023_44177_MOESM1_ESM.pdf]

Dissecting the genetic landscape of GPCR signaling through phenotypic profiling in *C. elegans*

Longjun Pu[1, 2, 3¶], Jing Wang[1, 2,3¶], Qiongxuan Lu[1, 2, 3], Lars Nilsson[1, 2, 3], Alison Philbrook[4], Anjali Pandey[4], Lina Zhao[1, 2, 3], Robin Van Schendel[5], Alan Koh[6, 7], Tanara V Peres[6, 7], Weheliye H Hashi[6, 7], Si Lhyam Myint[1, 8, 9], Chloe Williams[10], Jonathan D Gilthorpe[10], Sun Nyunt Wai[1, 8, 9], Andre Brown[6, 7], Marcel Tijsterman[5], Piali Sengupta[4], Johan Henriksson[1, 8, 11*], Changchun Chen[1, 2, 3*]

Supplementary Fig. 1 – Fig. 7

Supplementary Fig. 1

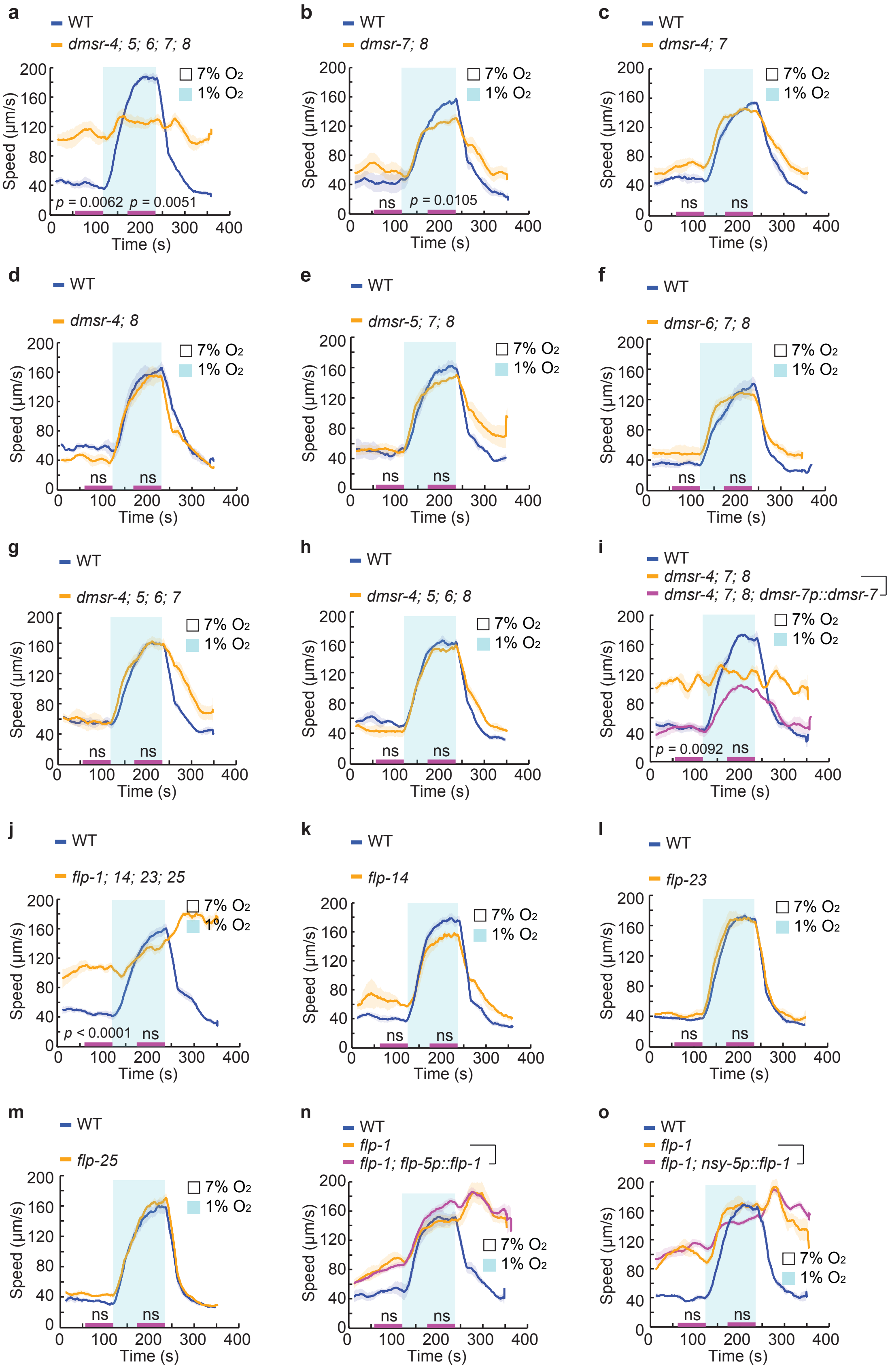

Supplementary Fig. 1: FLP-1 acts on DMSR-4, DMSR-7 and DMSR-8 to modulate hypoxia evoked locomotory responses

a–o Locomotory responses to rapid shifts from 7% O_2 to 1% O_2 of animals with indicated genotypes. Purple bars on X-axis indicate the time intervals used for statistical analysis. Data are presented as mean values +/- SEM. *p* values are displayed in individual plots. ns = not significant. Two-tailed Welch's *t* test. The number of independent assays for each strain is as follows: In (**a**), WT (n = 3) and *dmsr-4; dmsr-5; dmsr-6; dmsr-7; dmsr-8* quintuple mutants (n = 3). In (**b**), WT (n = 3) and *dmsr-7; dmsr-8* double mutant (n = 4). In (**c**), WT (n = 3) and *dmsr-4; dmsr-7* double mutant (n = 5). In (**d**), WT (n = 4) and *dmsr-4; dmsr-8* double mutant (n = 3). In (**e**), WT (n = 3) and *dmsr-5; dmsr-7; dmsr-8* triple mutant (n = 5). In (**f**), WT (n = 3) and *dmsr-6; dmsr-7; dmsr-8* triple mutant (n = 4). In (**g**), WT (n = 3) and *dmsr-4; dmsr-5; dmsr-6; dmsr-7* quadruple mutant (n = 3). In (**h**), WT (n = 3) and *dmsr-4; dmsr-5; dmsr-6; dmsr-8* quadruple mutant (n = 3). In (**i**), WT (n = 4), *dmsr-4; dmsr-7; dmsr-8* triple mutants (n = 3), and transgenic *dmsr-4; dmsr-7; dmsr-8* expressing *dmsr-7* genomic DNA from its endogenous promoter (n = 3). In (**j**), WT (n = 6) and *flp-1; flp-14; flp-23; flp-25* quadruple mutant (n = 5). In (**k**), WT (n = 4) and *flp-14* (n = 4). In (**l**), WT (n = 3) and *flp-23* (n = 3). In (**m**), WT (n = 3) and *flp-25* (n = 3). In (**n**), WT (n = 4), *flp-1* (n = 4), and transgenic *flp-1* expressing *flp-1* cDNA from *flp-5* promoter in RMG neurons (n = 3). In (**o**), WT (n = 3), *flp-1* (n = 4), and transgenic *flp-1(yum104)* expressing *flp-1* cDNA from *nsy-5* promoter in a set of sensory and interneurons, but not in AVK neurons (n = 3).

Supplementary Fig. 2

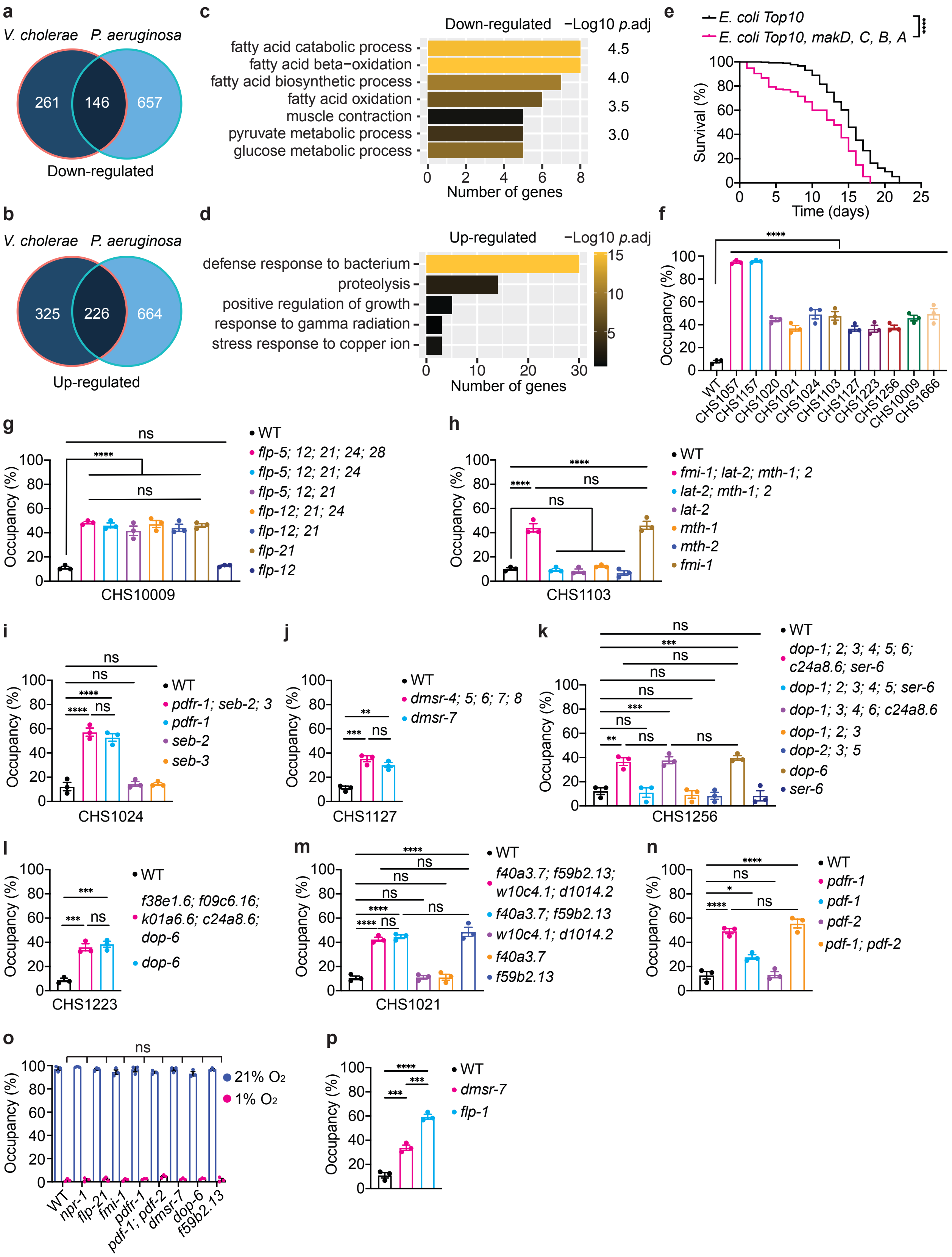

Supplementary Fig. 2: The identification of GPCR and neuropeptide genes that are required for the avoidance of *V. cholerae*

a, b Venn diagram displaying significantly down- (**a**) and up- (**b**) regulated genes in exposure to *V. cholerae* and *P. aeruginosa*. **c, d** GO categories for down- (**c**) and up- (**d**) regulated genes in response to *V. cholerae* with adjusted p <1e-20. **e** Survival curves of WT animals on *E. coli* strain Top10 and on Top10 strain expressing *makA*, *makB*, *makC*, and *makD* genes from *V. cholerae*. Data were generated from 3 biological replicates. **** = $p < 0.0001$. log-rank test. **f** Strains defective in escape from *V. cholerae*. Data were generated from 3 biological replicates. **** = $p < 0.0001$. One-way ANOVA, Tukey's multiple comparison. **g–m** The identification of relevant genes in strains CHS10009 (**g**), CHS1103 (**h**), CHS1024 (**i**), CHS1127 (**j**), CHS1256 (**k**), CHS1223 (**l**), and CHS1021 (**m**). The percentage of animals on pathogen lawn after 24 hours of *V. cholerae* exposure were plotted. Data were generated from 3 biological replicates. **** = $p < 0.0001$, *** = $p < 0.001$, ** = $p < 0.01$, and ns = not significant. One-way ANOVA, Tukey's multiple comparison. **n** The percentage of animals on pathogen lawn after 24 hours of *V. cholerae* exposure in animals with indicated genotypes. Data were generated from 3 biological replicates. **** = $p < 0.0001$, * = $p < 0.05$, and ns = not significant. One-way ANOVA, Tukey's multiple comparison. **o** Lawn occupancy of animals with indicated genotypes in 21% O_2 or 1% O_2 for 24 hours. The data were generated from 3 biological replicates. ns = no significant. Two-tailed *t* test. **p** The percentage of animals on pathogen lawn after 24 hours of *V. cholerae* exposure in WT, *dmsr-7(yum5087)*, and *flp-1(yum104)*. Data were generated from 3 biological replicates. **** = $p < 0.0001$ and *** = $p < 0.001$. One-way ANOVA, Tukey's multiple comparison. In **f–p**, data are presented as mean values +/- SEM. Source data are provided as a Source Data file. The exact p values are displayed in the Source Data file.

Supplementary Fig. 3

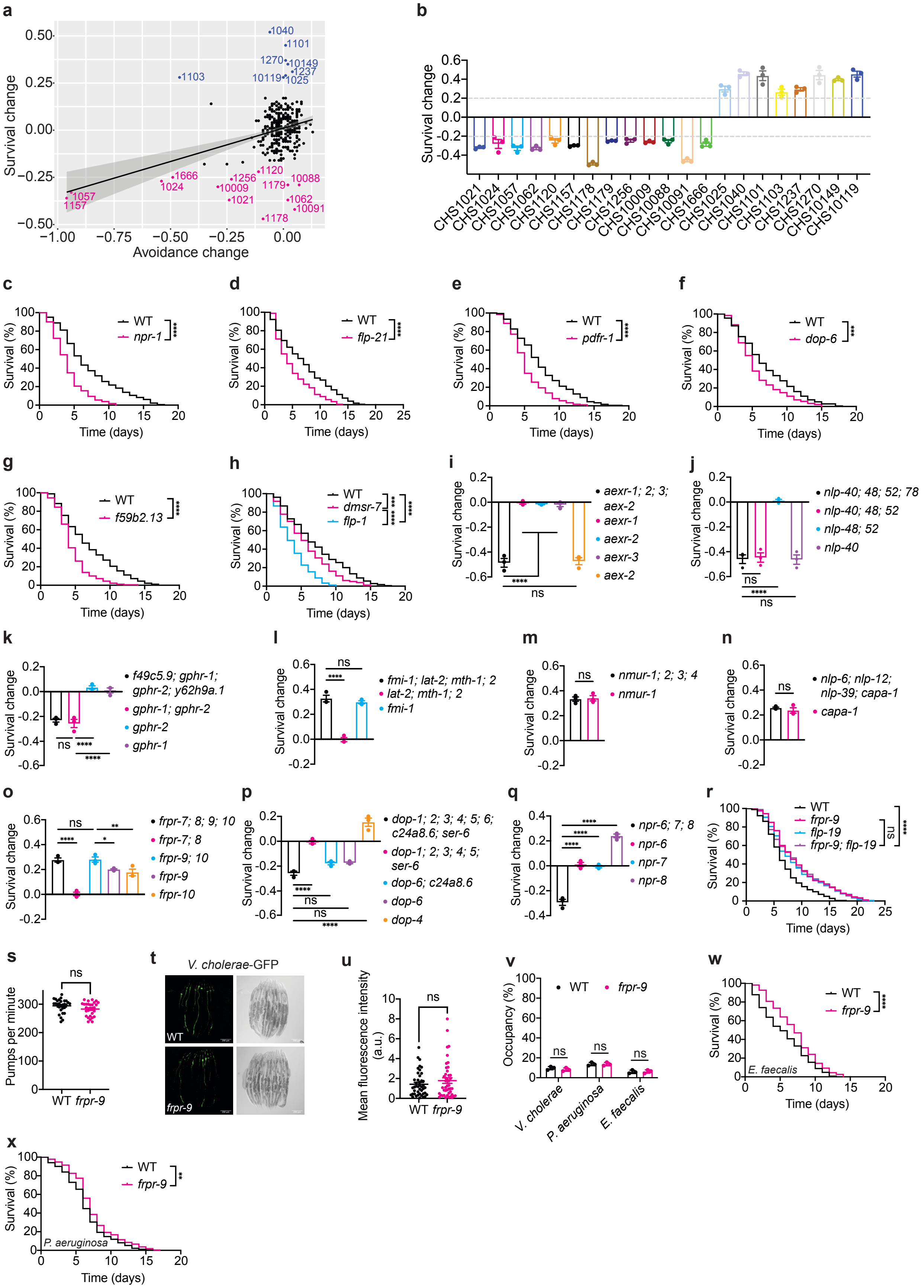

Supplementary Fig. 3: The identification of GPCR and neuropeptide genes that are required for the survival upon *V. cholerae* exposure

a The correlation between pathogen avoidance and animals' survival upon pathogen exposure ($\rho=0.28$). The hypersensitive and resistant strains for further analyses were indicated in magenta or blue, respectively. **b** The survival of the strains indicated in (**a**) on *V. cholerae*. **c–h** Survival curves of animals with indicated genotypes on *V. cholerae*. **** = $p < 0.0001$, *** = $p < 0.001$. log-rank test. **i–q** The identification of gene disruption that led to altered sensitivity to *V. cholerae* in the strains CHS1178 (**i**), CHS10091 (**j**), CHS1179 (**k**), CHS1103 (**l**), CHS1040 (**m**), CHS10149 (**n**), CHS1025 (**o**), CHS1256 (**p**), and CHS1062 (**q**). **** = $p < 0.0001$, ** = $p < 0.01$, * = $p < 0.05$, and ns = not significant. One-way ANOVA, Tukey's multiple comparison in (**i**, **j**, **k**, **l**, **o**, **p** and **q**), and two-tailed *t* test in (**m** and **n**). **r** Survival curves of animals with indicated genotypes on *V. cholerae*. **** = $p < 0.0001$ and ns = not significant. log-rank test. **s** Pharyngeal pumping per minute of WT and *frpr-9(yum1004)* mutants. n=30 of each strain. ns = not significant. Two-tailed *t* test. **t** Representative micrographs showing animals exposed to GFP labeled *V. cholerae* for 16 hours. **u** Quantitative analysis of fluorescence intensity in (**t**). n = 43 worms (WT) and n = 44 worms (*frpr-9*). ns = not significant. Two-tailed *t* test. **v** The percentage of animals on pathogen lawn after 24 hours of exposure. The strain genotypes used in this analysis were WT and *frpr-9(yum1004)*. ns = not significant. Two-tailed *t* test. **w** and **x** Survival curves of WT and *frpr-9(yum1004)* animals on *E. faecalis* OG1RF (**w**) or on *P. aeruginosa* PA14 (**x**). ** = $p < 0.01$, **** = $p < 0.0001$. log-rank test. In **c–h** and **r**, data were generated from 2 biological replicates. In **b** and **i–q,** data were generated from 3 biological replicates. Data are presented as mean values +/- SEM. Source data are provided as a Source Data file. The exact *p* values are displayed in the Source Data file.

Supplementary Fig. 4

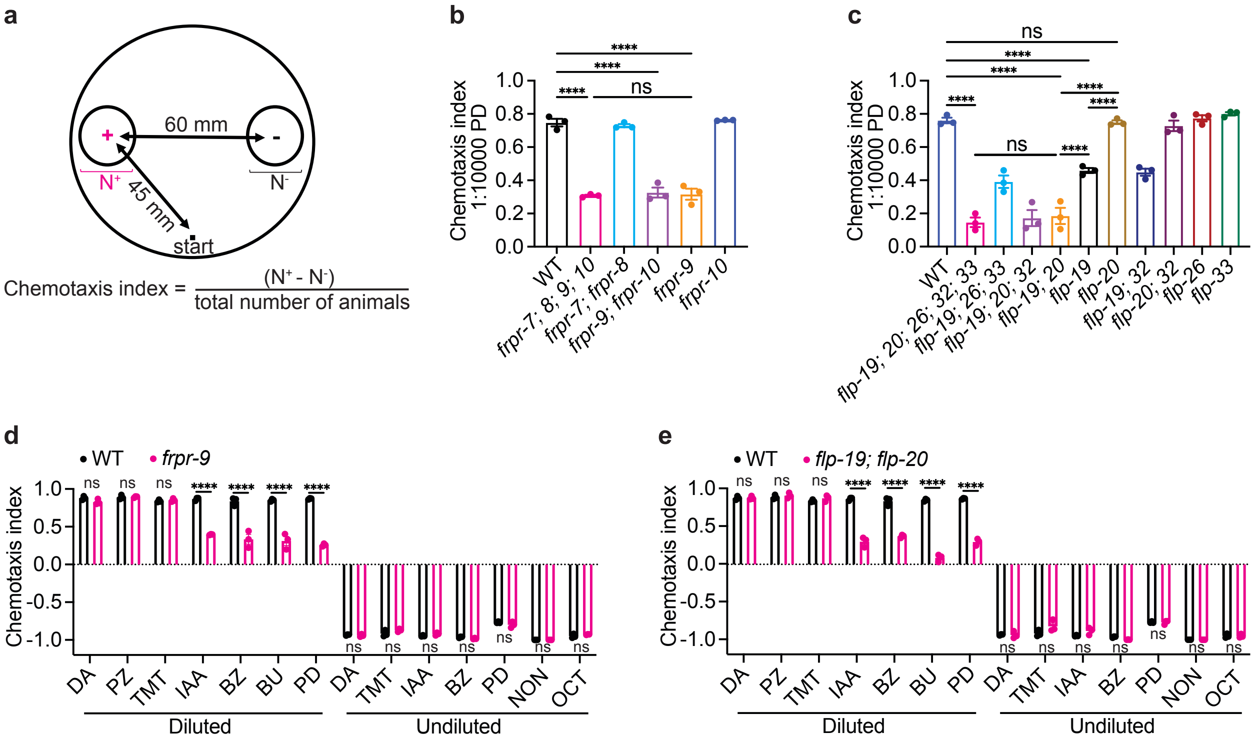

Supplementary Fig. 4: The identification of FRPR-9, FLP-19 and FLP-20 that are required for AWC-mediated chemosensation

a Schematic drawing of plate format in population assays to evaluate the chemotaxis of neuropeptide and neuropeptide receptor mutants. **b** The identification of the gene that was required for chemotaxis to 1:10000 2,3-pentanedione (PD) in the strain CHS1025: *frpr-7(yum1193); frpr-8(yum1194); frpr-9(yum1195); frpr-10(yum1196)* mutants. **** = $p < 0.0001$ and ns = not significant. One-way ANOVA, Tukey's multiple comparison. **c** The identification of the gene that was required for chemotaxis to 1:10000 2,3-pentanedione (PD) in the strain CHS10063: *flp-19(yum457); flp-20(yum403); flp-26(yum499); flp-32(yum404); flp-33(yum498)* mutants. **** = $p < 0.0001$ and ns = not significant. One-way ANOVA, Tukey's multiple comparison. **d** Chemotaxis indices of WT and *frpr-9(yum1004)* to various diluted and undiluted odorants. **** = $p < 0.0001$ and ns = not significant. Two-tailed *t* test. **e** Chemotaxis indices of WT and *flp-19(yum1005); flp-20(yum1006)* to various diluted and undiluted odorants. **** = $p < 0.0001$ and ns = not significant. Two-tailed *t* test. In all figure panels, data were generated from 3 biological replicates. Data are presented as mean values +/- SEM. Source data are provided as a Source Data file. The exact *p* values are displayed in the Source Data file.

Supplementary Fig. 5

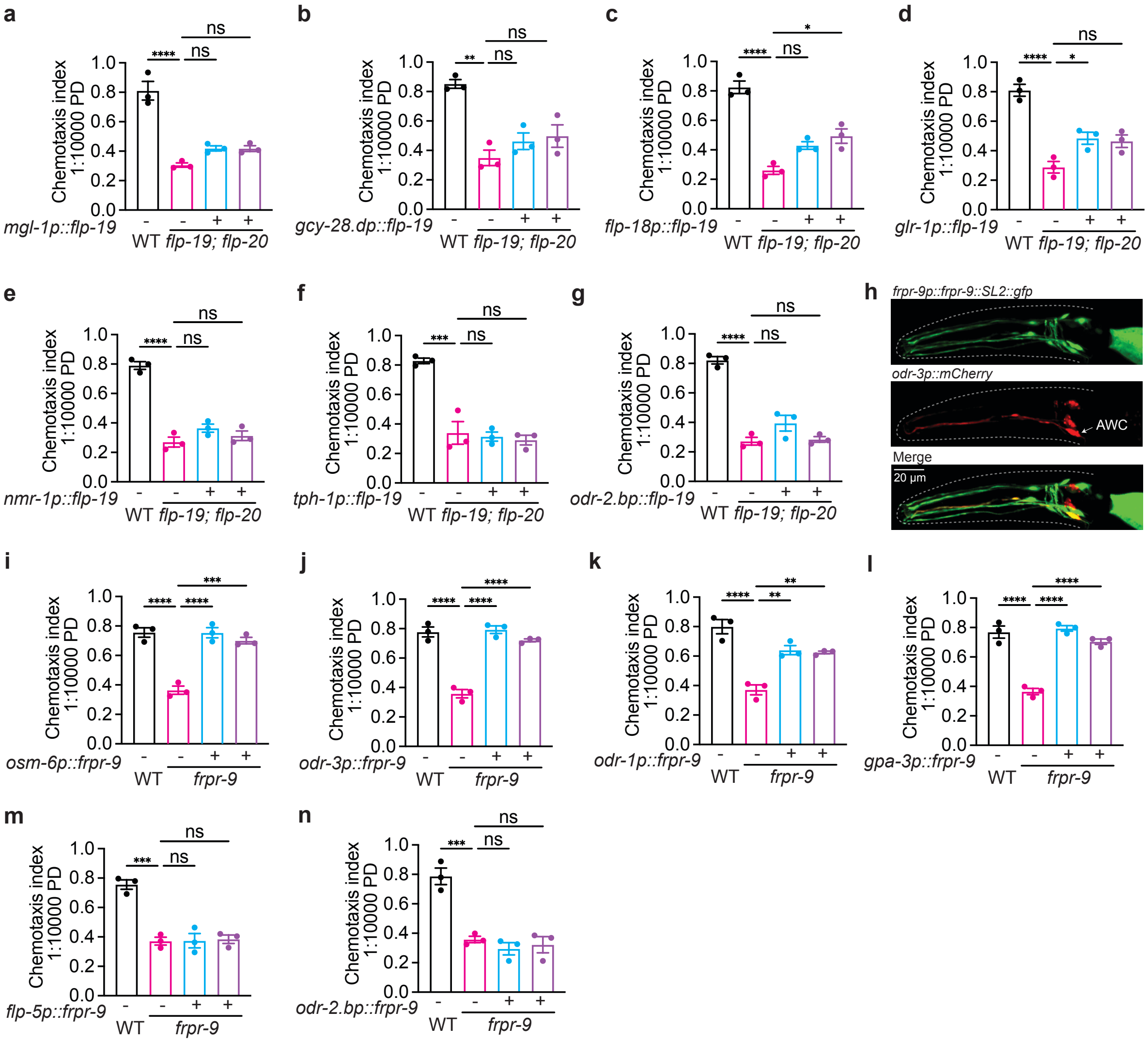

Supplementary Fig. 5: Peptidergic signaling is required for AWC-mediated chemosensation

a–g Chemotaxis indices to 1:10000 diluted 2,3-pentanedione (PD) of animals with indicated genotypes: WT, *flp-19(yum1005); flp-20(yum1006)* and two independent lines of transgenic *flp-19(yum1005); flp-20(yum1006)* expressing *flp-19* cDNA under *mgl-1* promoter (**a**), *gcy-28.d* promoter (**b**), *flp-18* promoter (**c**), *glr-1* promoter (**d**), *nmr-1* promoter (**e**), *tph-1* promoter (**f**) and *odr-2.b* promoter (**g**). **** = $p < 0.0001$, *** = $p < 0.001$, ** = $p < 0.01$, * = $p < 0.05$, and ns = not significant. One-way ANOVA, Tukey's multiple comparison. **h** Representative image showing GFP expression from a *frpr-9p::frpr-9::SL2::gfp* polycistronic construct, and mCherry expression from a *odr-3p::mCherry* polycistronic construct. **i-n** Chemotaxis indices to 1:10000 diluted 2,3-pentanedione (PD) of animals with indicated genotypes WT, *frpr-9(yum1004)* and two independent lines of transgenic *frpr-9(yum1004)* expressing *frpr-9* genomic DNA under *osm-6* promoter (**i**), *odr-3* promoter (**j**), *odr-1* promoter (**k**), *gpa-3* promoter (**l**), *flp-5* promoter (**m**), and *odr-2.b* promoter (**n**). **** = $p < 0.0001$, *** = $p < 0.001$, ** = $p < 0.01$, and ns = not significant. One-way ANOVA, Tukey's multiple comparison. In all chemotaxis assays, data were generated from 3 biological replicates. Data are presented as mean values +/- SEM. Source data are provided as a Source Data file. The exact *p* values are displayed in the Source Data file.

Supplementary Fig. 6

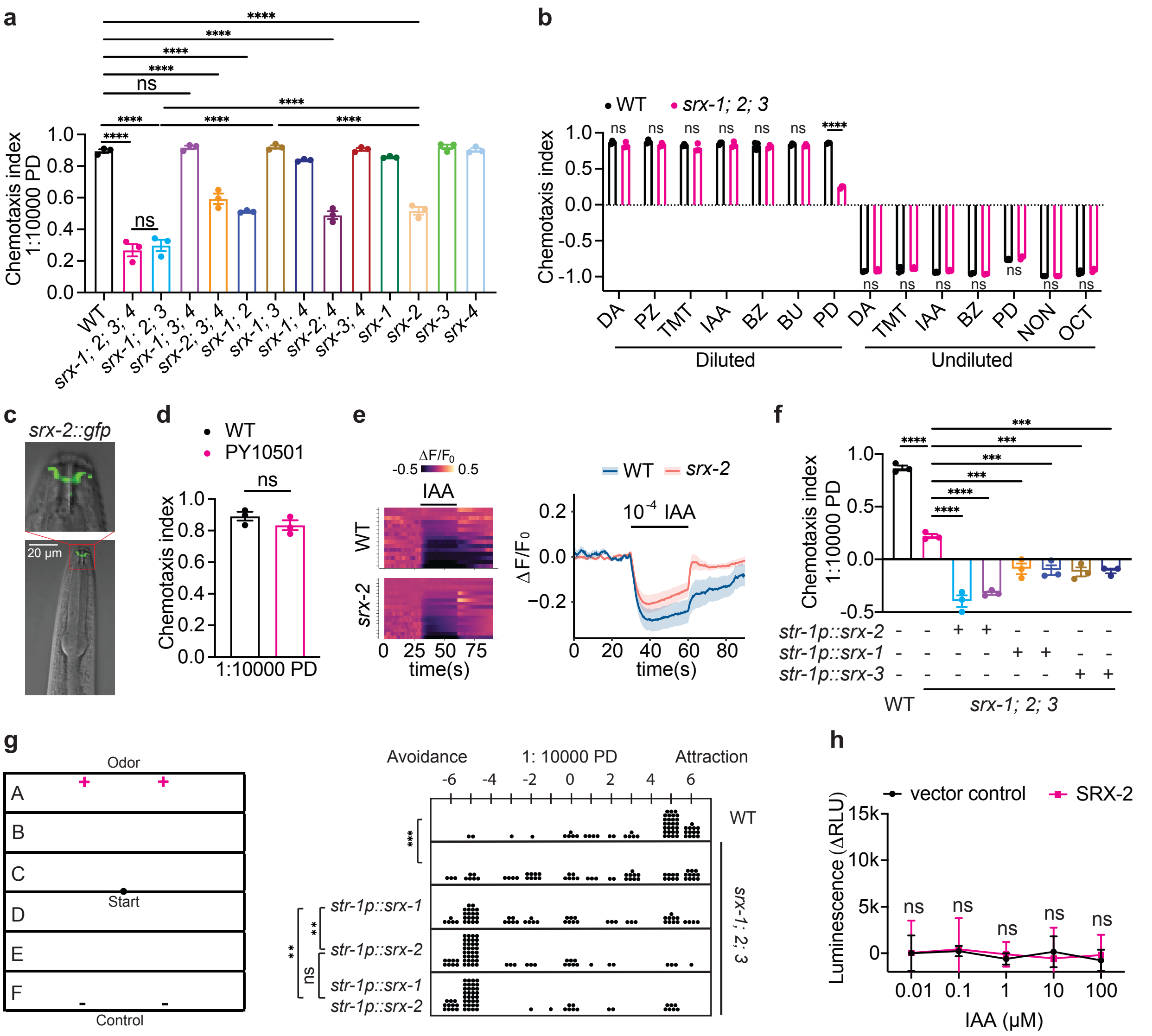

Supplementary Fig. 6: The identification of SRX-1, SRX-2 and SRX-3 as the putative receptors for 2,3-pentanedione

a The identification of the gene that was required for chemotaxis to 1:10000 2,3-pentanedione (PD) in the strain CHS1135. Data were generated from 3 biological replicates. **** = $p < 0.0001$ and ns = not significant. One-way ANOVA, Tukey's multiple comparison. **b** Chemotaxis indices of animals with indicated genotypes to the diluted and undiluted odorants. Data were generated from 3 biological replicates. **** = $p < 0.0001$ and ns = not significant. Two-tailed *t* test. **c** Representative image showing the endogenous SRX-2-GFP expression in the cilia. **d** Chemotaxis indices of WT and the calcium imaging strain to 1:10000 2,3-pentanedione (PD). Data were generated from 3 biological replicates. ns = not significant. Two-tailed *t* test. **e** Heatmap (left) and average values (right) of GCaMP3 fluorescence intensity changes to 10^{-4} IAA in AWC^{OFF} neurons of WT and *srx-2(yum1007)* mutant. **f** Chemotaxis indices to 1:10000 diluted 2,3-pentanedione (PD) of animals with indicated genotypes. *srx-1, srx-2*, or *srx-3* cDNA was expressed from *str-1* promoter in AWB neurons. Data were generated from 3 biological

replicates. *** = $p < 0.001$, **** = $p < 0.0001$. One-way ANOVA, Tukey's multiple comparison. **g** Left: schematic drawing of plate format used in single worm chemotaxis assays. Right: scores to 1:10000 diluted 2,3-pentanedione (PD) of animals with indicated genotypes. *srx-1, srx-2 or srx-3* cDNA was expressed from *str-1* promoter in AWB neurons. Each dot indicates the score of one animal. Data were generated from 3 biological replicates, each with 20 worms. ** = $p < 0.01$, *** = $p < 0.001$, ns=not significant. Two-sided Mann-Whitney rank sum test. **h** Intracellular cAMP concentrations in response to different dilutions of IAA in SRX-2 (red) and in vector (black) transfected cells. Data were generated from 3 biological replicates, each with 3 technical replicates. ns = not significant. Two-tailed *t* test. In **a**, **b**, **d**, **f**, and **h**, data are presented as mean values +/- SEM. Source data are provided as a Source Data file. The exact *p* values are displayed in the Source Data file.

Supplementary Fig. 7

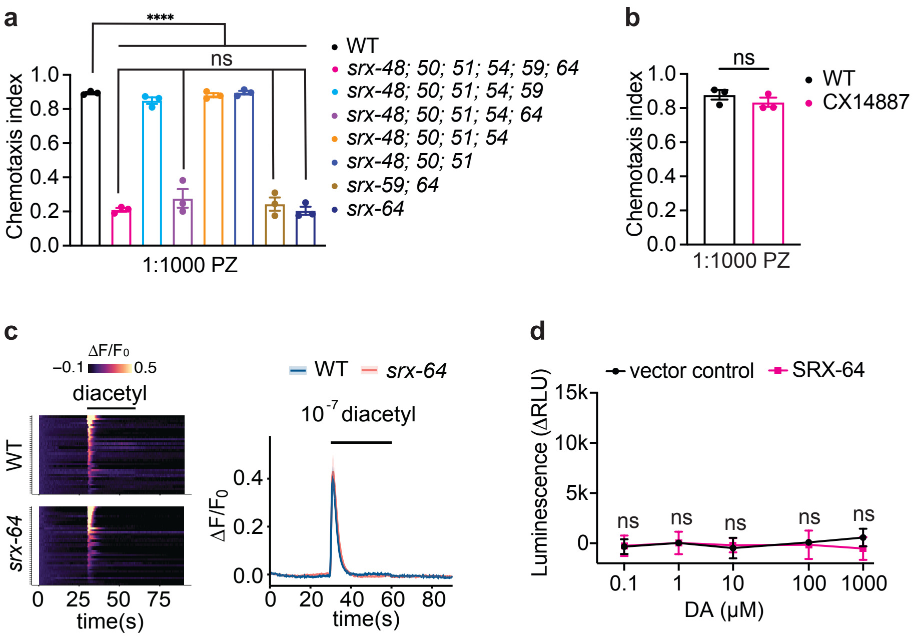

Supplementary Fig. 7: The identification of SRX-64 as the putative receptor for pyrazine

a The identification of the gene that was required for chemotaxis to 1:1000 pyrazine in the strain CHS1146: *srx-48(yum1857); srx-50(yum1858); srx-51(yum1859); srx-54(yum1860); srx-59(yum1862); srx-64(yum1863)* mutants. Data were generated from 3 biological replicates. **** = $p < 0.0001$ and ns = not significant. One-way ANOVA, Tukey's multiple comparison. **b** Chemotaxis indices of WT and calcium imaging strain CX14887 kyIs598 [*gpa-6::GCaMP2.2b*] to 1:1000 pyrazine (PZ). Data were generated from 3 biological replicates. ns = not significant. Two-tailed *t* test. **c** On the left, heatmap displays GCaMP2 fluorescence intensity changes to 10^{-7} diacetyl in AWA neurons of both WT and *srx-64(yum1002)*. Each row on the heatmap represents the response of one AWA neuron. On the right, average fluorescence intensity changes in AWA neurons in response to a 30 second pulse of 10^{-7} diacetyl in WT and *srx-64(yum1002)*. **d** Intracellular cAMP concentrations in response to different dilutions of diacetyl (DA) in pcDNA3-HA-SRX-64 (red) and in pcDNA3 vector transfected cells (black). Data were generated from 3 biological replicates, each with 3 technical replicates. ns = not significant. Two-tailed *t* test. In all figure panels, data are presented as mean values +/- SEM. Source data are provided as a Source Data file. The exact *p* values are displayed in the Source Data file.
