# Supplementary material for: Dissecting the genetic landscape of GPCR signaling through phenotypic profiling in C. elegans
Source: Nat Commun. 2023 Dec 18;14:8410. doi: 10.1038/s41467-023-44177-z (PMC10728192; doi:10.1038/s41467-023-44177-z)
Supplement: Supplementary file 3 — Description of Additional Supplementary Files [file 41467_2023_44177_MOESM3_ESM.pdf]

Description of Additional Supplementary Files

File name: Supplementary Data 1

Description: CRISPR/Cas9-mediated genome editing of GPCR-encoding genes.

a The list of 1675 GPCR-encoding genes as well as 29 non-GPCR-encoding genes (at the bottom of the table) included in this study. **b** The list of 126 unannotated chemoreceptor GPCRs. Six members of the *srw* subfamily, which are closely related to neuropeptide receptors, are highlighted in blue. **c** The cell type description for individual neurons in *C. elegans*. **d** The detailed sequence information of crRNA (guide), ssODN and genotyping primers used for the genome-editing of GPCR encoding genes. The restriction enzyme used for genotyping each targeted site is indicated. The genome-editing of three genes *lin-17*, *mom-5*, and *lat-1* was unsuccessful, and eighteen genes highlighted in yellow have not been attempted. In addition, twenty-nine non-GPCR encoding genes were also disrupted in the mutant strains. **e** The list of 284 GPCR mutant strains in our collection, of which 278 strains were genome-sequenced. **f** Genome sequencing information of the total of 1725 targeted sites in 1628 GPCR-encoding genes and 29 other genes. The genome sequencing reads were compared to the pre-designed ssODN. The comparisons are not listed if the genome sequencing reads match perfectly with ssODN. The mismatches are indicated in red in genome sequencing reads and ssODN sequences, and are further highlighted with (?) in the comparison column. The large deletions, insertions, or other types of mismatches are described in the 'comments' column.

File name: Supplementary Data 2

Description: CRISPR/Cas9-mediated genome editing of neuropeptide encoding genes

a The detailed sequence information of crRNA (guide), ssODN and genotyping primers used for the genome-editing of 152 neuropeptide encoding genes. The restriction enzyme used for genotyping each targeted site is indicated. **b** The list of 38 neuropeptide mutant strains in our collection.

File name: Supplementary Data 3

Description: Hypoxia-evoked locomotory responses of GPCR and neuropeptide mutants

Locomotory responses to rapid shifts from 7% O_2 to 1% O_2 of each strain in the GPCR and neuropeptide mutant libraries. The locomotory speed at 1% O_2 was scored and denoted using the '+' or '-' symbols. Explanations for the symbols and additional comments are placed at the bottom of the table.

File name: Supplementary Data 4

Description: The responses of GPCR and neuropeptide mutants to the infection of *V. cholerae*

a The log2 fold change in gene expression after an 8-hour exposure to *V. cholerae* when compared to the control animals on OP50 lawn. Synchronized L4 animals were used. Five independent RNA samples were prepared for both OP50 and *V. cholerae* treatments. Both adjusted and unadjusted *p* values are displayed. Two-sided Wald test. **b** The behavioral avoidance of GPCR and neuropeptide mutants to *V.*

cholerae exposure. The number of animals remained on the pathogen lawn was counted after 24 hours of pathogen exposure. The avoidance index was calculated as (the number of animals out of the pathogen lawn / Total number of animals). The avoidance index change was computed as (avoidance index of the mutant – avoidance index of wild type) / avoidance index of wild type. 30 synchronized L4 animals were used in each assay, and each strain was assayed at least twice. **c** The survival of each strain was scored every day. The mean survival days were calculated. The data displayed were the survival changes, which were computed as (mean survival days of the mutant – mean survival days of wild type) / mean survival days of wild type. The same worm population of each strain was used for both avoidance and survival assays. 30 synchronized L4 animals were used in each assay, and each strain was assayed at least twice.

File name: Supplementary Data 5

Description: The chemotaxis of GPCR and neuropeptide mutants to a panel of volatile attractants and repellents

a Chemotaxis indices of GPCR and neuropeptide mutants to the diluted odorants 1:2000 diacetyl (DA), 1:1000 pyrazine (PZ), 1:2000 2, 4, 5-trimethylthiazole (TMT), 1:200 isoamyl alcohol (IAA), 1:1000 benzaldehyde (BZ), 1:10000 2-butanone (BU), and 1:10000 2,3-pentanedione (PD), and the undiluted diacetyl (DA), 2, 4, 5-trimethylthiazole (TMT), isoamyl alcohol (IAA), benzaldehyde (BZ), 2,3-pentanedione (PD), 2-nonanone (NON), and 1-octanone (OCT). Each strain was assayed twice, and 150–200 synchronized day-one adults were used in each assay. **b** The Pearson correlation score between neuropeptide and neuropeptide receptor mutants in response to both odorants and *V. cholerae*. Two-sided *t* test.

File name: Supplementary Data 6

Description: Strains used in this study

This table lists all the other strains, which are not included in the GPCR and neuropeptide mutant libraries.

File name: Supplementary Data 7

Description: Other reagents used in this study

a All the plasmids used in this study. **b** The cloning primers and the other genome-editing sequences that are not used in the generation of mutant libraries are listed here. **c** The other reagents or resource, including bacterial strains, antibodies, chemicals, commercial assay kits, and cell lines, are listed.
