# Supplementary material for: Dissecting the genetic landscape of GPCR signaling through phenotypic profiling in C. elegans
Source: Nat Commun. 2023 Dec 18;14:8410. doi: 10.1038/s41467-023-44177-z (PMC10728192; doi:10.1038/s41467-023-44177-z)
Supplement: Supplementary file 11 — Reporting Summary [file 41467_2023_44177_MOESM11_ESM.pdf]

nature portfolio

Corresponding author(s): Johan Henriksson, Changchun Chen

Last updated by author(s): Jun 20, 2023

Reporting Summary

Nature Portfolio wishes to improve the reproducibility of the work that we publish. This form provides structure for consistency and transparency in reporting. For further information on Nature Portfolio policies, see our Editorial Policies and the Editorial Policy Checklist.

Statistics

For all statistical analyses, confirm that the following items are present in the figure legend, table legend, main text, or Methods section.

n/a	Confirmed	
☐	☒	The exact sample size (*n*) for each experimental group/condition, given as a discrete number and unit of measurement
☐	☒	A statement on whether measurements were taken from distinct samples or whether the same sample was measured repeatedly
☐	☒	The statistical test(s) used AND whether they are one- or two-sided<br>*Only common tests should be described solely by name; describe more complex techniques in the Methods section.*
☐	☒	A description of all covariates tested
☐	☒	A description of any assumptions or corrections, such as tests of normality and adjustment for multiple comparisons
☐	☒	A full description of the statistical parameters including central tendency (e.g. means) or other basic estimates (e.g. regression coefficient) AND variation (e.g. standard deviation) or associated estimates of uncertainty (e.g. confidence intervals)
☐	☒	For null hypothesis testing, the test statistic (e.g. *F*, *t*, *r*) with confidence intervals, effect sizes, degrees of freedom and *P* value noted<br>*Give P values as exact values whenever suitable.*
☒	☐	For Bayesian analysis, information on the choice of priors and Markov chain Monte Carlo settings
☒	☐	For hierarchical and complex designs, identification of the appropriate level for tests and full reporting of outcomes
☒	☐	Estimates of effect sizes (e.g. Cohen's *d*, Pearson's *r*), indicating how they were calculated

Our web collection on statistics for biologists contains articles on many of the points above.

Software and code

Policy information about availability of computer code

Data collection	Nikon NIS elements software for image collection
Data analysis	Genome sequencing analysis: Picard (version 2.19.2) (http://broadinstitute.github.io/picard), Pindel (version 0.2.5a8), and IGV (version 2.16.1).<br>Hypoxia video analysis: home-made MatLab program Zentracker (https://github.com/wormtracker/zentracker)<br>RNAseq analysis: STAR v2.7.9a, featureCounts v2.0.3, DESeq2<br>Images: ImageJ Fiji (https://imagej.net/software/fiji/)

For manuscripts utilizing custom algorithms or software that are central to the research but not yet described in published literature, software must be made available to editors and reviewers. We strongly encourage code deposition in a community repository (e.g. GitHub). See the Nature Portfolio guidelines for submitting code & software for further information.

Data

Policy information about availability of data

All manuscripts must include a data availability statement. This statement should provide the following information, where applicable:

- Accession codes, unique identifiers, or web links for publicly available datasets
- A description of any restrictions on data availability
- For clinical datasets or third party data, please ensure that the statement adheres to our policy

The WGS sequencing data is available on ArrayExpress #E-MTAB-12983 [https://www.ebi.ac.uk/biostudies/arrayexpress/studies/E-MTAB-12983], and the RNA-seq data at ArrayExpress #E-MTAB-13001 [https://www.ebi.ac.uk/biostudies/arrayexpress/studies/E-MTAB-13001]. Source data are provided with this paper.

Research involving human participants, their data, or biological material

Policy information about studies with human participants or human data. See also policy information about sex, gender (identity/presentation), and sexual orientation and race, ethnicity and racism.

Reporting on sex and gender	Not applicable to this study
Reporting on race, ethnicity, or other socially relevant groupings	Not applicable to this study
Population characteristics	Not applicable to this study
Recruitment	Not applicable to this study
Ethics oversight	Not applicable to this study

Note that full information on the approval of the study protocol must also be provided in the manuscript.

Field-specific reporting

Please select the one below that is the best fit for your research. If you are not sure, read the appropriate sections before making your selection.

☒ Life sciences ☐ Behavioural & social sciences ☐ Ecological, evolutionary & environmental sciences

For a reference copy of the document with all sections, see nature.com/documents/nr-reporting-summary-flat.pdf

Life sciences study design

All studies must disclose on these points even when the disclosure is negative.

Sample size	Sample sizes for all analyses are determined following established protocols and standard practice in C. elegans.
Data exclusions	No data was excluded.
Replication	In the main and supplementary figures, at least two biological replicates, each with at least three technical replicates, were included in the assays. In all large-scale screens, each strain was assayed twice. If the data from two replicates differ, a third assay was included.
Randomization	Synchronized worms were randomly distributed into experimental and control groups for each genotype
Blinding	Blinding was applied to all the assays whenever possible. Certain strains exhibited obvious locomotory phenotypes, which made blinded analysis difficult.

Reporting for specific materials, systems and methods

We require information from authors about some types of materials, experimental systems and methods used in many studies. Here, indicate whether each material, system or method listed is relevant to your study. If you are not sure if a list item applies to your research, read the appropriate section before selecting a response.

Materials & experimental systems

n/a	Involved in the study
☐	☒ Antibodies
☐	☒ Eukaryotic cell lines
☒	☐ Palaeontology and archaeology
☐	☒ Animals and other organisms
☒	☐ Clinical data
☒	☐ Dual use research of concern
☒	☐ Plants

Methods

n/a	Involved in the study
☒	☐ ChIP-seq
☒	☐ Flow cytometry
☒	☐ MRI-based neuroimaging

Antibodies

Antibodies used	HA (rabbit polyclonal, AGRISERA, cat# AS12 2220), Alexa Fluor™ 568 Secondary Antibody Donkey Anti-Rabbit (Invitrogen, Cat# A10042)
Validation	The commercial antibody was validated by its manufacturer and titrated in the lab to determine its optimal concentration. Validation statements and experiments can be obtained from the following websites (https://www.agrisera.com/en/artiklar/ha-tag.html).

Eukaryotic cell lines

Policy information about cell lines and Sex and Gender in Research

Cell line source(s)	HEK293T cells (ATCC, CRL-11268)
Authentication	HEK293T was authenticated by ATCC with no further authentication in the lab.
Mycoplasma contamination	Mycoplasma free.
Commonly misidentified lines (See ICLAC register)	No commonly misidentified cell lines were used in this study.

Animals and other research organisms

Policy information about studies involving animals; ARRIVE guidelines recommended for reporting animal research, and Sex and Gender in Research

Laboratory animals	Caenorhabditis elegans hermaphrodite in L4 or day one adult stages were used in this study. GPCR library strains, neuropeptide library strains, and the other strains used in this study are provided in Supplementary Tables 1, 2 and 6, respectively. All C. elegans strains used in the study are available from the authors or from the standard commercial source: Caenorhabditis Genetic Center (CGC, https://cgc.umn.edu/).
Wild animals	No wild animals were used in this study.
Reporting on sex	Sex was not considered in the study design.
Field-collected samples	No field-collected samples were used in the study.
Ethics oversight	No ethical approval was required.

Note that full information on the approval of the study protocol must also be provided in the manuscript.
